# Integrin-Mediated Tumorigenesis and Its Therapeutic Applications

**DOI:** 10.3389/fonc.2022.812480

**Published:** 2022-02-11

**Authors:** Qingling Li, Ting Lan, Jian Xie, Youguang Lu, Dali Zheng, Bohua Su

**Affiliations:** ^1^Fujian Key Laboratory of Oral Diseases, Fujian Provincial Engineering Research Center of Oral Biomaterial, School and Hospital of Stomatology, Fujian Medical University, Fuzhou, China; ^2^Department of Preventive Dentistry, School and Hospital of Stomatology, Fujian Medical University, Fuzhou, China

**Keywords:** integrin, cancer, signaling transduction, talin, FAK

## Abstract

Integrins, a family of adhesion molecules generally exist on the cell surface, are essential for regulating cell growth and its function. As a bi-directional signaling molecule, they mediate cell-cell and cell-extracellular matrix interaction. The recognitions of their key roles in many human pathologies, including autoimmunity, thrombosis and neoplasia, have revealed their great potential as a therapeutic target. This paper focuses on the activation of integrins, the role of integrins in tumorigenesis and progression, and advances of integrin-dependent tumor therapeutics in recent years. It is expected that understanding function and signaling transmission will fully exploit potentialities of integrin as a novel target for tumors.

## Introduction

Integrins are a type I transmembrane protein and the main ligands for cell adhesion. There are altogether 18 α and 8 β subunits known in mammals, generating 24 kinds of heterodimers (1). Each subunit has a large ectodomain, a single transmembrane domain (TMD) and a comparatively short cytoplasmic tail. The transmembrane region is the key link of information transmission and interaction between TMD and cytoplasmic tail, regulating the affinity between integrins and their ligands. Though they vary in size, the classic α subunit is made up of around 1,000 amino acids, compared with 750 for the β subunit (2).

As unique adhesion molecules, integrin can signal in both directions across the plasma membrane. Intracellular activators like talins trigger the conformational changes of integrins and recruit multivalent protein complexes (“clusting”) that bind directly or indirectly to the integrin cytoplasmic tail (3–5). These combinations represent a complex, highly dynamic system that relates to ligand-binding affinity, which is responsible for regulating various aspects of cellular fate like cell migration and extracellular matrix (ECM) assembly and remodeling (6). Events introduced above are called “inside-out” signaling. Integrins also enable human cells to respond to changes in the extracellular environment through outside-in signaling. Outside information communicates to cells *via* intracellular means, bringing about changes in cell polarity, cytoskeletal structure, gene expression, cell survival and proliferation (7).

Integrin heterodimers are often classified by the special sequences they can recognize. Those sequences are generally known as RGD or LDV tripeptides, or some complex peptide like GFOGER. Researchers conventionally classified integrins into 4 types: RGD receptors, collagen receptors, laminin receptors and leukocyte-specific receptors (8). For example, Integrin αvβ3 binds to a spectrum of ECM molecules using the RGD triple-peptide motif (9), which includes von Willebrand factor, fibronectin, fibrinogen, proteolyzed forms of collagen and laminin, and vitronectin. Other integrins, like α5β1, can only selectively bind to fibronectin (10).

The binding of integrins and ligands are not only located in the classical extracellular matrix (ECM). Integrin interacts with various proteins on the surfaces of cells, even on fungal cells and viruses. Those proteins include hormones, growth factors, and polyphenols (11). Notably, many growth factors bind to the ECM, and the spatial arrangement of integrin and growth factor binding sites in the ECM enables simultaneous engagement of their cognate receptors on the plasma membrane (12). Integrin involves in proliferative signaling, tumor invasion and metastasis, evasion of apoptosis, and stimulation of angiogenesis. This was achieved by cooperating with growth factor receptors like epidermal growth factor receptor (EGFR), ErbB-2 to amplify downstream pathways such as PI3K, AKT, MAPK and the Rho family small GTPases (13). Tejeshwar et al. found that EGFR regulates integrin tension and the spatial organization of focal adhesions, and that the mechanical tension threshold for outside-in integrin activation is tunable by EGFR (14). There are also plenty of non-ECM molecules that interact with integrins, making integrins essential mediators of cell biology.

## Activation and Signal Transmission of Integrin

**Figure 1:**
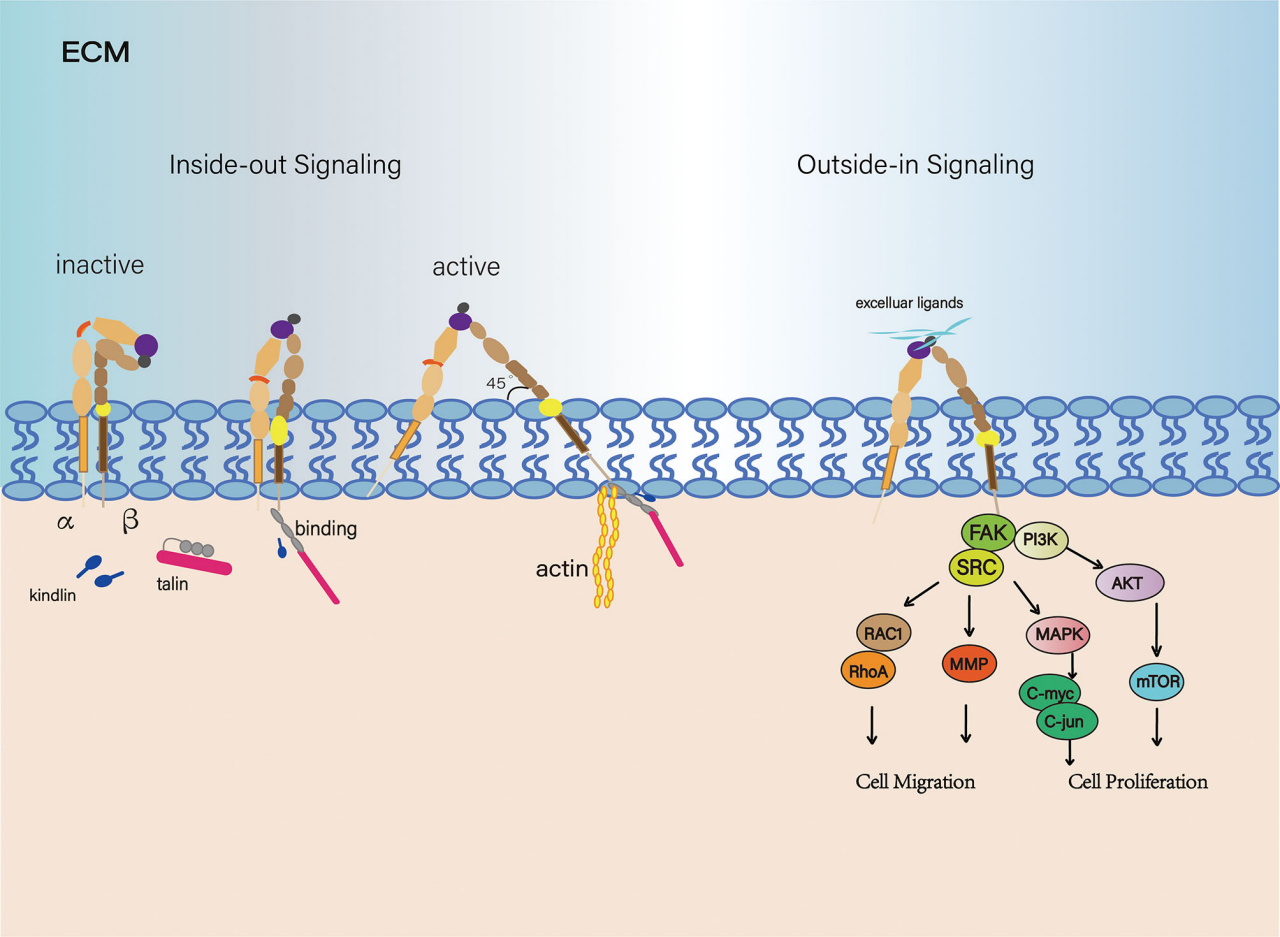
Signal transduction of the integrin family. From an inactive conformation to a low affinity, intermediate state that may arise from talin and/or kindlin binding. And in the active state, integrin subunits were separated, forming a 45 degree angle. Integrins are connected to the actin cytoskeleton and can initiate cytoskeletal remodeling (Left). Integrin-controlled cell migration is largely mediated by signaling pathways involving members of the focal adhesion kinase (FAK)-SRC family kinase. Integrins are ligated and initiate multiple downstream effectors.

**Figure 2:**
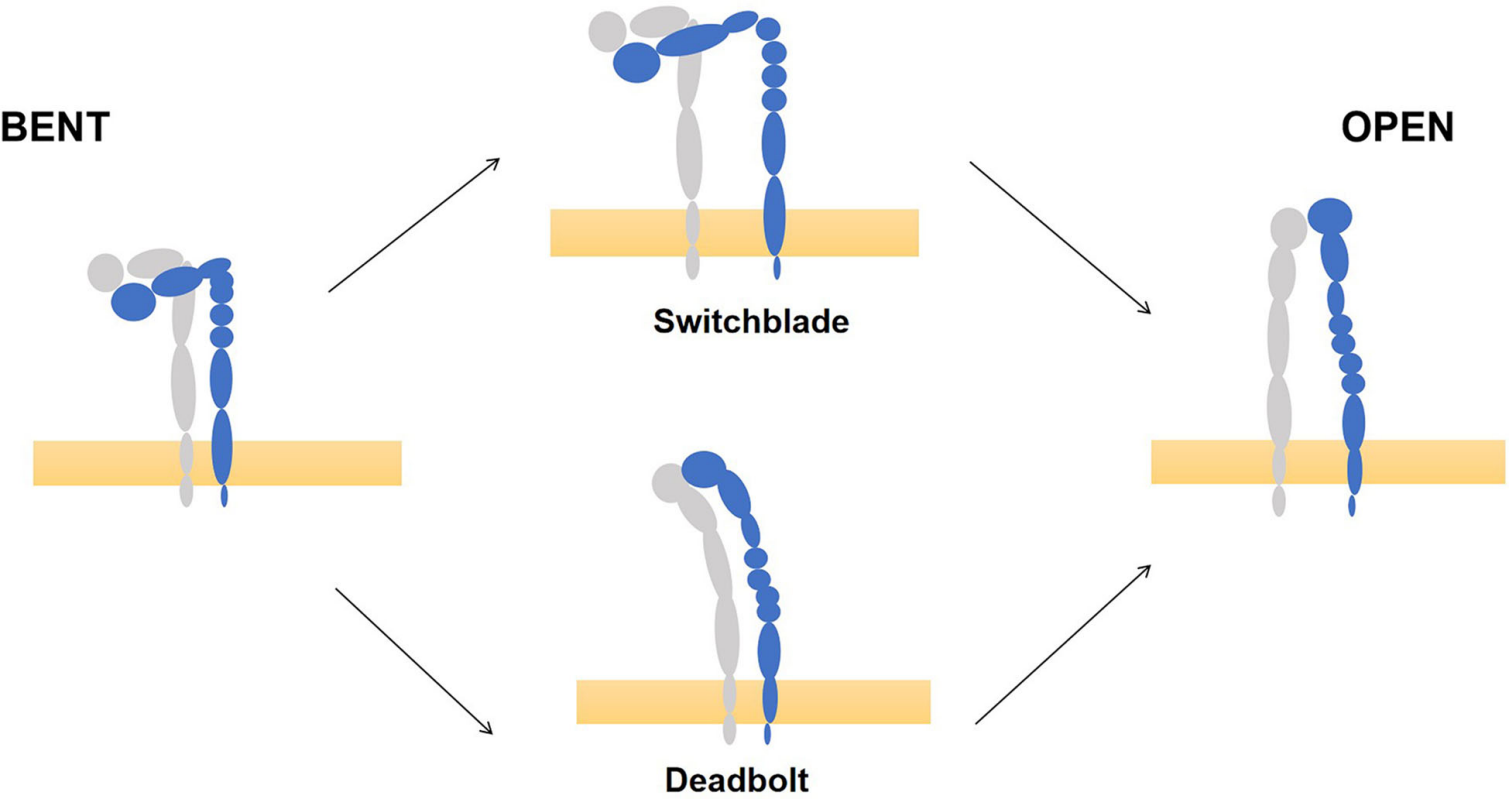
Models of integrin conformational activation. Take integrin αVβ3 for example, there are two common models for activation of integrins, the ‘‘switchblade’’ and the ‘‘deadbolt’’ models, to describe a transition state from the low affinity state (left) and high affinity state (right). (This picture modified from Bidone TC. Coarse-Grained Simulation of Full-Length Integrin Activation. Biophys J. 2019 Mar 19;116 (6):1000-1010. doi: 10.1016/j.bpj.2019.02.011. Epub 2019 Feb 22.).

Each integrin exists either in the "bent" state of low-affinity or in an extended high-affinity conformation (15–17). The transition from a "bent" to an extended conformation is called "activation," which is reversible and rapid. This process involves two key mechanisms: the extension of the head and the separation of the legs, which are triggered by "inside-out" or "outside-in" signals (18, 19). However, recent work clearly illustrated that integrins are vertically positioned on the cell membrane and exist in three main conformations: bent-closed (inactive), extended-closed (active, low affinity) and extended-open (active, high affinity) conformations (20). (**Figure 1**) There are two common models for activation of integrins: the ‘‘switchblade’’ and the ‘‘deadbolt’’ models, which describe a transition state from the curved one to the extended conformation (21–23). (**Figure 2**)

As we know, one of the fully studied integrin pathways is the focal adhesion kinase (FAK) signaling pathway. Upon binding to its specific ligand, it leads to maximal FAK activation. The FAK-Src complex has multiple downstream effectors (24). FAK-Src complex promotes the activity of a GTPase which belongs to the Ras superfamily, which is generally known as Rac1 (Ras-related C3 botulinum toxin substrate 1). Rac1 activation is involved in spreading and in the early stages of migration (25). At later stages of cell spreading or for instance, by constitutive activation of αvβ3 *via* ligand binding, RhoA activity leads to the formation of stressfibers and promotes migration (26). In addition, phosphorylation of FAK leads to the Ras-mediated activation of the MAP-kinase pathway (MAPK/ERK pathway), which is associated with proliferation and tumorigenic behavior. Through this pathway, several transcription factors such as the oncogene C-myc and C-jun are activated *via* phosphorylation. Therefore, the activation of the MAPK pathway leads to the transcription of genes that are important for cell proliferation and cell cycle progression. This pathway can be activated by cell adhesion (e.g., binding of α5β1 to fibronectin) or growth factors (such as epidermal growth factor (EGF) (27, 28). Moreover, phosphorylated FAK connects with PI3K, which leads to the activation of AKT *via* PDK1 (29). The AKT signaling pathway can also lead to the phosphorylation of YAP which acts as an apoptotic suppressor (30). The activation of YAP represents a cross-talk with a newer signaling pathway known as Hippo pathway. This pathway controls organ size by regulating cell proliferation and apoptosis (31).

Dynamic remodeling of adhesions is an important mechanism employed by cells to regulate integrin–ECM interactions and cellular signaling. This is done through rapid endocytic and exocytic trafficking of integrin receptors during cell migration, invasion and cytokinesis. Integrin traffic is relevant in several pathological processes, especially in cancer. Importantly, conceptual progress in the field has identified well-known cancer oncogenes and mutations as being crucial regulators of integrin traffic. To support their proliferation rate, cancer cells exploit active integrin-mediated ECM endocytosis to directly acquire nutrients from the extracellular environment (32).

Integrin activation is a process of conformational changes which allows integrins to bind their ligands. This process is well modulated through the interaction between the integrin α/β cytoplasmic tails (CTs) and their binding partners. Many researchers believe that the change of cytoplasmic tail is the main cause of conformational change (33). Evidence suggests that talins and kindlins are the proteins that bind to cytoplasmic domain and mediate this process (34). In "inside-out" signaling, intracellular activators such as talins or kindlins, binding to the CTs of β subunit leads to the separation of the α and β tails and induces conformational changes in the ectodomain, thereby increasing its affinity for ligands, also known as the "activation" of integrin (35, 36). Conformational changes and clustering of a single integrin can affect affinity to its ligands (15). The affinity of integrin can also be regulated by ligand *in vitro* to induce conformational changes in the extracellular domain of integrin. Studies suggest that intracellular tensile forces can also lead to integrin activation that is ultrasensitive to lower levels of forces compared with cytoskeletal adaptor binding alone (37). In general, the bi-directional signaling reactions are regulated by the dynamic interaction of integrins and proteins on both sides of the membrane.

Talin is one of the most well-known integrin activators that mediates integrin adherence to the extracellular matrix. Talins activate integrins by binding to the CTs of β-integrin *via* its typical 4.1-protein/ezrin/radixin/moesin (FERM) domain. The membrane-proximal NPxY of β-tail has been identified as the talin-binding site, and the membrane-distal NPxY specifically interacts with kindlins. By binding integrins to actin, talin increases the affinity to the corresponding ligands (integrin activation) as well as recruits a large number of proteins to form the core of the integrin adhesion complex, which in turn activates adhesion plaque kinases (FAK) and Src family kinases (SFKs) (**Figure 1**). For example, loss of talin-1 leads to diminished *in vivo* metastasis of prostate cancer cells *via* FAK–Src complexes and AKT kinase signaling (38). Downregulation of talin-1 has also been shown to promote hepatocellular carcinoma progression (39). In platelets, talin-1 is the principal direct effector of Rap1 GT Pases that regulates platelet integrin activation in hemostasis (40). Researchers now have established a pipeline approach to evaluate the effect of talin-1 mutations. Through a series of computational methods, biochemical and cell biological analysis, results suggested cancer-related point mutations in talin-1 can affect cell behaviour and so may contribute to cancer progression (41).

Another family of FERM-containing proteins is kindlins, which are a recently discovered integrin interaction partners that play a synergistic role in talin activation of integrin. Although the molecular details of talin-mediated integrin activation are known, the mechanism of kindlin involvement in this process remains elusive. In the knockout and overexpression experiments, kindlin-1, kindlin-2, and kindlin-3 could regulate specific integrin activation, but only in accordance with the interaction between talin-1 and the cytoplasmic tail of integrin. Activation of integrin αIIbβ3 was enhanced by co-expression of kindlin-1 or kindlin-2 and decreased by knocking out endogenous Si-RNA of kindlin-2. The ligand binding to integrin αIIbβ3 is activated due to an overexpressed N-terminal head domain of talin (42–44). Ussar S, et al. found that deletion of kindlin-1 in intestinal epithelial cells or colon cancer cell lines reduced talin-dependent integrin β1 activation or directly reduced integrin-mediated cell adhesion (45). Interrupting kindlins’ dimer formation impairs kindlin-mediated integrin activation (46). Zainab H. used all-atomic microsecond-scale molecular dynamics simulations of integrin aIIbβ3 TM/CT structure in an explicit lipid-water environment and then found that kindlin-2 cooperates with talin-1 to facilitate integrin aIIbβ3 activation by enhancing talin-1 interaction with the membrane proximal (MP) region of β3-integrin (47). Both talins and kindlins are essential for integrin conformational activation, to which they seem to contribute differently by allowing the vinculin-mediated perception of mechanical forces (talins) and triggering biochemical signaling pathways (kindlins) (48), e.g. through paxillin and focal adhesion kinase (FAK) (49–51). Though they cooperatively support integrin activation, the functional significance of post-translational modifications of kindlins controlling integrin signaling has been gradually recognized (52).

When it comes to integrin activation, some accelerants such as paxillin (51, 53), ADAP (54) and migfillin (55) must be mentioned. There are also some inhibitors, such as ICAP-1 (56), filamin (57, 58) and sharpin (59), allowing for forfine-tuning of the integrin activation process. Findings showed that sharpin may complex with both kindlin-1 and the integrin-β1 cytoplasmic tail to restrict the talin head domain binding, thus inhibiting β1-integrin activation. Besides, integrins also interact with many cytoplasmic proteins, such as Filamina, Dok1 and 14-3-3 proteins, etc. (60).

## Effects of Integrins on Self-Renewal and Proliferation of Tumor Stem Cells

In cancer, the strict control of proliferation is lost due to extrinsic factors such as the presence of mitogenic compounds (growth factors, cytokines or exogenous substances) or intrinsic factors such as activation of oncogenes, converting cancer cells in a self-sufficient entity. In this context, integrins play a crucial role by directly promoting proliferation or by indirectly interacting with growth factor receptors. Interactions between growth factor receptors and integrins in cancer are involved in proliferation. Three types of interactions can be distinguished (1): direct interaction (2), modulation of expression levels and (3) reciprocal activation (61). Integrin signaling has been shown to drive many stem cell functions. Plaks et al. found that specialized extracellular matrix niches and integrin signaling support the function of normal stem cells and their tumor derivatives (62). It has been found that integrin β1 mediates the adhesion of basal keratinocytes to the basement membrane in epithelium and controls the stem cell renewal by regulating the polarity axis of asymmetric cell and cell cycle progression (63). The highly expressed laminin binding to integrin α6β1 of tumor stem cells not only promotes adhesion to the surface of the endothelial basement membrane near the lumen, but also transmits self-renewal signals through FAK (64). Integrin αVβ5 could play as a functional cancer stem cell marker essential for glioblastoma maintenance and ZIKV infection, providing potential brain tumor therapy (65). A recent study found arsenic and BaP co-exposure human bronchial epithelial cells have a high expression of integrin α4, leading to activation of the Hedgehog pathway and PI3K/Akt pathway, enhancing arsenic and BaP co-exposure-induced cancer stem cell (CSC)-like property and tumorigenesis (66).

## Role of Integrins in Adhesion and Tumor Invasion

Extensive evidence shows that the expression of integrin is significantly different in tumor cells compared to normal ones. Integrin signaling in cancer cells is dysfunctional, which is of significance to understand how tumor cells use integrin activity to regulate invasion and movement and to study the regulatory mechanism of integrin function.

It is well known that the transition from carcinoma *in situ* to invasive cancer is driven by a series of adhesion changes. By remodeling or dissolving E-cadherin-dependent junctions and integrin-mediated adhesion, unparted cancer cells or groups of cancer cells would separate from adjacent normal cells and the basement membrane below. Through FAK and SFKs, integrins directly phosphorylate E-cadherin-β-catenin complex to remodeling E-cadherin-dependent junctions, promoting the migration and invasion of cancer cells (67). Integrin-mediated adhesion of fibronectin triggers a negative feedback signal that blocks the formation of E-cadherin mediated cell-to-cell adhesion (68). Putting integrin β1 into β1-deficient epithelial cells resulted in loss of cell contact and dispersion of cells (69), suggesting that integrin-extracellular matrix adhesion plays an inhibitory role in the regulation of cell-cell junctions. Therefore, the internal and external signals of integrins can disrupt intercellular adhesion by increasing myosins’ contractibility and E-cadherin junction stability through FAK and SRC signals (70). Integrin and integrin-dependent processes are implicated in almost every step of cancer development, including tumor growth, invasion and perfusion into the vascular system, survival of circulating tumor cells, extravasation into secondary sites, and metastasis and colonization of new tissues. Integrins expressed on the cell surface is to adhere to the ECM. Ligation provides traction that is essential tumor cell survival and invasion. A recent study has indicated that hypoxia selectively enhances the expression of integrin α5β1 receptor in breast cancer to promote metastasis (71). The expression and potential roles of thrombospondins (TSP-4) in the crosstalk between CAFs and gallbladder cancer (GBC) cells has remained unclear. Research showed that a complex TSP-4/integrin α2/HSF1/TGF-β cascade mediates reciprocal interactions between GBC cells and CAFs, providing a promising therapeutic target for gallbladder cancer patients (72).

For most solid tumors, the basement membrane first needs to be breached. This process is thought to require proteolysis, and integrins play their roles by upregulating the expression of matrix metalloproteinases (MMP) and promoting the activation and function of proteinases at the extracellular matrix. Integrins control cell migration and invasion by influencing the activity and localization of matrix-degrading proteases, such as urokinase-type plasminogen activator (uPA) and MMP2 (73, 74) Invasive cancers penetrate the stroma through a variety of different integrin-dependent mechanisms and migrate to surrounding tissues in the form of a single cell or groups of cells (75). Futhermore, tumor-associated fibroblasts (CAFs) can promote cancer progression through several integrin-related mechanisms. Invasion is caused by deposition or regulation of fiberectin arrangement or by direct physical pulling of cancer cells from the primary tumor (76–79). In order to metastasize smoothly, tumor cells must attach to vasculature in distant organs and penetrate into perivascular tissues. Thrombosis is thought to support cancer metastasis through the recruitment of fibronectin to activate integrins. After extravasation, the contact of integrin with the extracellular matrix in perivascular tissue could determine whether the inoculated tumor cell would continue to proliferate or become dormant state (80–82). Integrin trafficking is also crucial for collective cell migration or morphogenetic movements of cell sheets. Rab-coupling protein (RCP)-dependent integrin recycling pathway was employed by invasive cancer cells for effective migration (83, 84).

## Effects of Multiple Integrin Signals on Tumor Microenvironment

Generally, tissue has a strictly regulated, specific optimum hardness (85), which is perceived by cells through integrins and their cytoskeletons. Hence, integrins are important mechanical receptors, and together with other adherent proteins such as integrin-activated proteins, talin, nucin and CRK-related substrates, convert mechanical signals into biochemical signals (86, 87). Several studies have discussed the role of integrin in angiogenesis, especially the integrin αv. Evidence suggests that integrin αv promotes tumor angiogenesis, depending on environments (88). Integrin α6β4 may also exert a similar environment-dependent pro-angiogenesis effect (89). In contrast, integrin α3β1 signaling in endothelial cells negatively regulates tumor angiogenesis by decreasing VEGFR2 expression (90). Signals from integrins also influence other behaviors in the tumor microenvironment. Studies show that TNFα pro-apoptotic signaling is regulated by the ECM and the integrin that is engaged, and Integrin α6β1 is inhibitory for the pro-apoptotic signal of TNF (91).

Integrins play bidirectional regulatory roles between cancer cells and cancer-associated fibroblasts (CAFs). CAFs that express IL-32 contain an RGD cell attachment sequence that binds to integrin β3-positive cancer cells to promote breast cancer cell invasion and metastasis (92). CAF-derived extracellular vesicles that express annexin A6 plays a pivotal role in gastric cancer drug resistance *via* activation of β1 integrin-FAK-YAP signaling (93). Colorectal cancer cells express integrin avβ6 activated CAFs through TGF-β, which subsequently secrete stromal cell-derived factor-1 (SDF-1) and promote colorectal cancer cell metastasis (94). These research studies reveal that integrins act as receptors that regulate the interactions between CAFs and cancer cells in tumor progression and drug resistance. Studies in the future may reveal more about the integrin signaling mechanisms involved about remodeling the tumor microenvironment during tumor development. Factors secreted by cancer cells profoundly alter the biology and composition of the stroma by inducing immune cells, triggering angiogenesis, and inducing the activation of CAFs, which generates a lot of tumor-promoting signals (76).

## Clinical Application of Integrin

Integrins have been seen as potential therapeutic targets since they were discovered to promote pathogenic processes. The inhibition of integrins has led to several marketed drugs, and many others are being investigated preclinically in both academic and industry settings. Since 2015, there have been at least 130 clinical trials of integrin-targeted therapies (95). Unfortunately, there are still a few unsuccessful inhibitors (**Table 1**). Efalizumab, which targeted αL integrins, was withdrawn from the market because of multiple cases of progressive multifocal leukoencephalopathy (PML), said to be involved with inhibition of α4-containing integrins and αLβ2 (96).

**Table 1:** Integrin-targeting drugs once came out.

Inhibitor Name	Target	Mechanism	Application	In Market
Lifitegrast	αLβ2	prevents lymphocyte adhesion	Dry eye disease	2016
Vedolizumab	α4β7	inhibits binding to MADCAM1	Ulcerative colitis and Crohn’s disease	2014
Natalizumab	Pan-α4	inhibits ligand binding to α4β7 and α4β1	Multiple sclerosis and Crohn’s disease	2004
Efalizumab	αL	preventing lymphocyte activation and migration	Plaque psoriasis	2003 (withdrawn 2009)
Tirofiban	αIIbβ3	inhibits binding to fibrinogen	Coronary syndrome and CVD	1998
Eptifibatide	αIIbβ3	inhibits binding to fibrinogen	Coronary syndrome and CVD	1998

Previous studies have found that α4 and β2 integrins are receptors mediating the neutrophil adhesion to the endothelium. Researchers evaluated the α4 and β2 integrins’ expression and functions in human primary neutrophils obtained from patients having chronic non-healing wounds and undergoing a prolonged hyperbaric oxygen therapy (150 kPa per 90 minutes). Cell adhesion function of both neutrophilic integrins α4β1 and β2 was significantly reduced, which could be of great importance for the design of novel therapeutic protocols focused on anti-inflammatory agents (97). Integrin αVβ3 is highly expressed on activated endothelial cells of tumor neovasculature and thus is key to tumor angiogenesis. RGD-binding integrins, mainly the αv integrin subfamily and important to the whole integrin family, are introduced about their expression in different human cancers and their pre-clinical antagonists. (**Table 2**) New molecules that target αv-containing integrins are now entering clinical trials for fibrotic diseases, including idiopathic pulmonary fibrosis (IPF) and nonalcoholic steatohepatitis (NASH), which have high and increasingly unmet medical need (95, 98, 99).

**Table 2:** αv-integrins expressed in different human cancers and their pre-clinical antagonists.

Integrin	Cancer Type	Main Expression Feature	Drug	Drug Targeted Cancer Type	Clinical Trial
αvβ3	Gastric cancer	Stroma and endothelia ↑, correlates with survival	Etaracizumab (Abegrin)	Colorectal/melanoma/prostate/thyroid cancer	Phase II
	Glioma	Correlates with grade	Intetumumab (CNTO 95)	Colorectal/melanoma/prostate/thyroid cancer	Phase II
	Lung cancer brain metastasis	Endothelia ↑ tumor cells ↓	Abciximab (c7E3)	Melanoma/breast cancer	Pre-clinical
	Non-small cell lung cancer	Endothelia ↑ tumor cells ↓	Vitaxin (MEDI-532)	Melanoma/breast cancer	Phase II
	Oral cancer	Intratumoral endothelia ↑	Cilengitide	Melanoma/breast cancer	Phase II
	Pancreatic cancer	Involved in lymph node metastasis	HM-3	Lung/liver/stomach cancer	Phase I
	Prostate cancer	Peritumor ↑	AP25	Melanoma/gastric/hepatic/breast carcinoma	Pre-clinical
αvβ5	Gastric cancer	Tumor cells, stroma and endothelial cells↑ independent prognostic factor in intestinal-type	Intetumumab (CNTO 95)	Melanoma/Prostate cancer	Phase II
	Lung cancer brain metastasis	Endothelia ↑ tumor cells ↓	Cilengitide	Melanoma/breast cancer	Pre-clinical
	Non-small cell lung cancer	Tumor and stroma cells ↑ no correlation with survival			
	Prostate cancer	Tumor and stroma cells ↑, no correlation with survival			
α5β1	Oral cancer	Stroma ↑	Volociximab (M200)	Melanoma/prostate cancer	Phase II
	Ovarian cancer	Correlates with survival	ATN-161	Glioblastoma	Phase II
αvβ6	Gastric cancer	Potential prognostic marker in early stage	Intetumumab (CNTO 95)	Prostate cancer/melanoma	Phase II
	Basal cell carcinoma	Infiltrative subtype ↑			
	Non-small cell lung cancer	Intratumoral Heterogeneity ↑			

↑ means up-regulation; ↓ means down-regulation.

Integrins can also be used in diagnostic imaging. Integrin-inhibiting peptide Apticitide (TC-99M-P280), a gpIIbIIIa imaging technique for the diagnosis of acute deep venious thrombosis, is now available. [99mTc]3PRGD2 imaging is valuable for the diagnosis and staging of esophageal cancer. It may be less sensitive than [18F]FDG imaging for detecting metastatic lesions in small lymph nodes. The T/B value was correlated with the expression of integrin αVβ3 (100). Integrin αVβ3 in imaging is in the PH2 trial phase. It is reported that other imaging agents are in the early stage of development (101, 102). As a PET tracer 18F-Alfatide II has been recently proven to possess good diagnostic value in distinguishing between breast cancer and benign breast lesions (103). Neil et. al found that Ga-68-Trivehexin is a promising probe for imaging of αVβ6-integrin expression in human cancers because of its high expression density at the boundary of tumor and healthy tissue (104). Recent studies also show that it may be possible to develop next-generation nanomedicine based on the combined derivatives of resveratrol and tetrac targeting the Integrin αvβ3 (105).

## Conclusion

Integrins have attracted much attention in recent years and are closely related to the development of cancers. We discussed much about the significance of integrin in cell migration and cell adhesion, which are important processes in tumor growth. Integrin-mediated cancer signals are also initiated by several integrin-binding proteins, which include talins, kindlins, MMPs, osteopontin, actinin and so on. Integrins interact with the actin cytoskeleton through these signaling molecules. And because of the polymerization and contraction generated by actin, the main signaling occurs while integrin activates. However, when integrin is misregulated, various mechanisms unfreeze the regulation of integrin signaling in cancer, enabling tumor cells to proliferate unrestrictedly and invade some tissue boundaries, allowing them to survive in microenvironments. The diversity of integrin and their roles in many diseases indicate the great potential of this superfamily as a drug target. Nowadays, designing drugs specific to integrin activation is possible as the structure of integrin has been recognized. By studying the mechanism of integrin and its related signaling pathways, we consider by regulating the expression of integrin or blocking the downstream signaling pathways of integrin to make its function. Although integrins have been discovered for more than 100 years, only a few of their inhibitors have been used in clinical applications, and no specific therapeutic inhibitors have been developed for cancer. Therefore, selectively blocking this acquired migration and invasion ability by targeting key metastatic molecules or regulatory proteins like integrin would be an attractive therapeutic strategy.

## Author Contributions

QL and TL reviewed the literature and drafted the article. JX, YL, DZ, and BS finalized the paper and provided suggestions to improve it. All authors participated in designing the concept of this manuscript. All authors contributed to the article and approved the submitted version.

## Conflict of Interest

The authors declare that the research was conducted in the absence of any commercial or financial relationships that could be construed as a potential conflict of interest.

## Publisher’s Note

All claims expressed in this article are solely those of the authors and do not necessarily represent those of their affiliated organizations, or those of the publisher, the editors and the reviewers. Any product that may be evaluated in this article, or claim that may be made by its manufacturer, is not guaranteed or endorsed by the publisher.

## References

[1] Hynes RO. Integrins: Bidirectional, Allosteric Signaling Machines. Cell (2002) 110(6):673–87. doi: 10.1016/S0092-8674(02)00971-6 12297042

[2] Campbell ID Humphries MJ. Integrin Structure, Activation, and Interactions. Cold Spring Harb Perspect Biol (2011) 3(3):a004994. doi: 10.1101/cshperspect.a004994 21421922 PMC3039929

[3] Horton ER Byron A Askari JA Ng DHJ Millon-Frémillon A Robertson J. Definition of a Consensus Integrin Adhesome and Its Dynamics During Adhesion Complex Assembly and Disassembly. Nat Cell Biol (2015) 17(12):1577–87. doi: 10.1038/ncb3257 PMC4663675 26479319

[4] Horton ER Humphries JD James J Jones MC Askari JA Humphries MJ. The Integrin Adhesome Network at a Glance. J Cell Sci (2016) 129(22):4159–63. doi: 10.1242/jcs.192054 PMC5117201 27799358

[5] Zaidel-Bar R Itzkovitz S Ma'ayan A Iyengar R Geiger B. Functional Atlas of the Integrin Adhesome. Nat Cell Biol (2007) 9(8):858–67. doi: 10.1038/ncb0807-858 PMC2735470 17671451

[6] Calderwood DA Campbell ID Critchley DR. Talins and Kindlins: Partners in Integrin-Mediated Adhesion. Nat Rev Mol Cell Biol (2013) 14(8):503–17. doi: 10.1038/nrm3624 PMC4116690 23860236

[7] Shattil SJ Kim C Ginsberg MH. The Final Steps of Integrin Activation: The End Game. Nat Rev Mol Cell Biol (2010) 11(4):288–300. doi: 10.1038/nrm2871 20308986 PMC3929966

[8] Humphries JD Byron A Humphries MJ. Integrin Ligands at a Glance. J Cell Sci (2006) 119(Pt 19):3901–3. doi: 10.1242/jcs.03098 PMC3380273 16988024

[9] Song Y Guo X Fu J He B Wang X Dai W. Dual-Targeting Nanovesicles Enhance Specificity to Dynamic Tumor Cells *In Vitro* and *In Vivo via* Manipulation of *α*v*β*3-Ligand Binding. Acta Pharm Sin B (2020) 10(11):2183–97. doi: 10.1016/j.apsb.2020.07.012 PMC7715539 33304785

[10] Kuonen F Surbeck I Sarin KY Dontenwill M Rüegg C Gilliet M. Tgfβ, Fibronectin and Integrin α5β1 Promote Invasion in Basal Cell Carcinoma. J Invest Dermatol (2018) 138(11):2432–42. doi: 10.1016/j.jid.2018.04.029 PMC6207534 29758283

[11] LaFoya B Munroe JA Miyamoto A Detweiler MA Crow JJ Gazdik T. Beyond the Matrix: The Many Non-ECM Ligands for Integrins. Int J Mol Sci (2018) 19(2):449. doi: 10.3390/ijms19020449 PMC5855671 29393909

[12] Cooper J Giancotti FG. Integrin Signaling in Cancer: Mechanotransduction, Stemness, Epithelial Plasticity, and Therapeutic Resistance. Cancer Cell (2019) 35(3):347–67. doi: 10.1016/j.ccell.2019.01.007 PMC6684107 30889378

[13] Stewart RL O'Connor KL. Clinical Significance of the Integrin α6β4 in Human Malignancies. Lab Invest (2015) 95(9):976–86. doi: 10.1038/labinvest.2015.82 PMC4554527 26121317

[14] Rao TC Ma VP Blanchard A Urner TM Grandhi S Salaita K. EGFR Activation Attenuates the Mechanical Threshold for Integrin Tension and Focal Adhesion Formation. J Cell Sci (2020) 133(13):jcs238840. doi: 10.1242/jcs.238840 32546532 PMC7358133

[15] Carman CV Springer TA. Integrin Avidity Regulation: Are Changes in Affinity and Conformation Underemphasized? Curr Opin Cell Biol (2003) 15(5):547–56. doi: 10.1016/j.ceb.2003.08.003 14519389

[16] Luo BH Carman CV Springer TA. Structural Basis of Integrin Regulation and Signaling. Annu Rev Immunol (2007) 25:619–47. doi: 10.1146/annurev.immunol.25.022106.141618 PMC1952532 17201681

[17] Ye F Kim C Ginsberg MH. Reconstruction of Integrin Activation. Blood (2012) 119(1):26–33. doi: 10.1182/blood-2011-04-292128 21921044 PMC3251231

[18] Shimaoka M Takagi J Springer TA. Conformational Regulation of Integrin Structure and Function. Annu Rev Biophys Biomol Struct (2002) 31:485–516. doi: 10.1146/annurev.biophys.31.101101.140922 11988479

[19] Salas A Shimaoka M Phan U Kim M Springer TA. Transition From Rolling to Firm Adhesion Can Be Mimicked by Extension of Integrin Alphalbeta2 in an Intermediate Affinity State. J Biol Chem (2006) 281(16):10876–82. doi: 10.1074/jbc.M512472200 PMC1599852 16505487

[20] Michael M Parsons M. New Perspectives on Integrin-Dependent Adhesions. Curr Opin Cell Biol (2020) 63:31–7. doi: 10.1016/j.ceb.2019.12.008 PMC7262580 31945690

[21] Takagi J Petre BM Walz T Springer TA. Global Conformational Rearrangements in Integrin Extracellular Domains in Outside-In and Inside-Out Signaling. Cell (2002) 110(5):599–11. doi: 10.1016/S0092-8674(02)00935-2 12230977

[22] Lefort CT Hyun YM Schultz JB Law FY Waugh RE Knauf PA. Outside-In Signal Transmission by Conformational Changes in Integrin Mac-1. J Immunol (2009) 183(10):6460–8. doi: 10.4049/jimmunol.0900983 PMC2860599 19864611

[23] Xiong JP Stehle T Goodman SL Arnaout MA. New Insights Into the Structural Basis of Integrin Activation. Blood (2003) 102(4):1155–9. doi: 10.1182/blood-2003-01-0334 12714499

[24] Horton ER Humphries JD Stutchbury B Jacquemet G Ballestrem C Barry ST. Modulation of FAK and Src Adhesion Signaling Occurs Independently of Adhesion Complex Composition. J Cell Biol (2016) 212(3):349–64. doi: 10.1083/jcb.201508080 PMC4739608 26833789

[25] Lawson CD Ridley AJ. Rho GTPase Signaling Complexes in Cell Migration and Invasion. J Cell Biol (2018) 217(2):447–57. doi: 10.1083/jcb.201612069 PMC5800797 29233866

[26] Huveneers S Danen EH. Adhesion Signaling - Crosstalk Between Integrins, Src and Rho. J Cell Sci (2009) 122(Pt 8):1059–69. doi: 10.1242/jcs.039446 19339545

[27] Wen S Hou Y Fu L Xi L Yang D Zhao M. Cancer-Associated Fibroblast (CAF)-Derived IL32 Promotes Breast Cancer Cell Invasion and Metastasis *via* Integrin β3-P38 MAPK Signalling. Cancer Lett (2019) 442:320–32. doi: 10.1016/j.canlet.2018.10.015 30391782

[28] Yee KL Weaver VM Hammer DA. Integrin-Mediated Signalling Through the MAP-Kinase Pathway. IET Syst Biol (2008) 2(1):8–15. doi: 10.1049/iet-syb:20060058 18248081

[29] Sun F Wang J Sun Q Li F Gao H Xu L. Interleukin-8 Promotes Integrin β3 Upregulation and Cell Invasion Through PI3K/Akt Pathway in Hepatocellular Carcinoma. J Exp Clin Cancer Res (2019) 38(1):449. doi: 10.1186/s13046-019-1455-x 31684995 PMC6829822

[30] Song G Ouyang G Bao S. The Activation of Akt/PKB Signaling Pathway and Cell Survival. J Cell Mol Med (2005) 9(1):59–71. doi: 10.1111/j.1582-4934.2005.tb00337.x 15784165 PMC6741304

[31] Meng Z Moroishi T Guan KL. Mechanisms of Hippo Pathway Regulation. Genes Dev (2016) 30(1):1–17. doi: 10.1101/gad.274027.115 26728553 PMC4701972

[32] Mana G Valdembri D Serini G. Conformationally Active Integrin Endocytosis and Traffic: Why, Where, When and How? Biochem Soc Trans (2020) 48(1):83–93. doi: 10.1042/BST20190309 32065228 PMC7054750

[33] Morse EM Brahme NN Calderwood DA. Integrin Cytoplasmic Tail Interactions. Biochemistry (2014) 53(5):810–20. doi: 10.1021/bi401596q PMC3985435 24467163

[34] Essex DW. Redox Control of Platelet Function. Antioxid Redox Signal (2009) 11(5):1191–225. doi: 10.1089/ars.2008.2322 19061441

[35] Wegener KL Campbell ID. Transmembrane and Cytoplasmic Domains in Integrin Activation and Protein-Protein Interactions (Review). Mol Membr Biol (2008) 25(5):376–87. doi: 10.1080/09687680802269886 PMC3000922 18654929

[36] Ginsberg MH Partridge A Shattil SJ. Integrin Regulation. Curr Opin Cell Biol (2005) 17(5):509–16. doi: 10.1016/j.ceb.2005.08.010 16099636

[37] Li J Springer TA. Integrin Extension Enables Ultrasensitive Regulation by Cytoskeletal Force. Proc Natl Acad Sci USA (2017) 114(18):4685–90. doi: 10.1073/pnas.1704171114 PMC5422820 28416675

[38] Sakamoto S McCann RO Dhir R Kyprianou N. Talin1 Promotes Tumor Invasion and Metastasis *via* Focal Adhesion Signaling and Anoikis Resistance. Cancer Res (2010) 70(5):1885–95. doi: 10.1158/0008-5472.CAN-09-2833 PMC2836205 20160039

[39] Chen P Lei L Wang J Zou X Zhang D Deng L. Downregulation of Talin1 Promotes Hepatocellular Carcinoma Progression Through Activation of the ERK1/2 Pathway. Cancer Sci (2017) 108(6):1157–68. doi: 10.1111/cas.13247 PMC5480078 28375585

[40] Lagarrigue F Paul DS Gingras AR Valadez AJ Sun H Lin J. Talin-1 Is the Principal Platelet Rap1 Effector of Integrin Activation. Blood (2020) 136(10):1180–90. doi: 10.1182/blood.2020005348 PMC7472713 32518959

[41] Azizi L Cowell AR Mykuliak VV Goult BT Turkki P Hytönen VP. Cancer Associated Talin Point Mutations Disorganise Cell Adhesion and Migration. Sci Rep (2021) 11(1):347. doi: 10.1038/s41598-020-77911-4 33431906 PMC7801617

[42] Montanez E Ussar S Schifferer M Bösl M Zent R Moser M. Kindlin-2 Controls Bidirectional Signaling of Integrins. Genes Dev (2008) 22(10):1325–30. doi: 10.1101/gad.469408 PMC2377186 18483218

[43] Ma YQ Qin J Wu C Plow EF. Kindlin-2 (Mig-2): A Co-Activator of Beta3 Integrins. J Cell Biol (2008) 181(3):439–46. doi: 10.1083/jcb.200710196 PMC2364684 18458155

[44] Harburger DS Bouaouina M Calderwood DA. Kindlin-1 and -2 Directly Bind the C-Terminal Region of Beta Integrin Cytoplasmic Tails and Exert Integrin-Specific Activation Effects. J Biol Chem (2009) 284(17):11485–97. doi: 10.1074/jbc.M809233200 PMC2670154 19240021

[45] Ussar S Moser M Widmaier M Rognoni E Harrer C Genzel-Boroviczeny O. Loss of Kindlin-1 Causes Skin Atrophy and Lethal Neonatal Intestinal Epithelial Dysfunction. PloS Genet (2008) 4(12):e1000289. doi: 10.1371/journal.pgen.1000289 19057668 PMC2585060

[46] Li H Deng Y Sun K Yang H Liu J Wang M. Structural Basis of Kindlin-Mediated Integrin Recognition and Activation. Proc Natl Acad Sci USA (2017) 114(35):9349–54. doi: 10.1073/pnas.1703064114 PMC5584418 28739949

[47] Haydari Z Shams H Jahed Z Mofrad MRK. Kindlin Assists Talin to Promote Integrin Activation. Biophys J (2020) 118(8):1977–91. doi: 10.1016/j.bpj.2020.02.023 PMC7175420 32191864

[48] Sun Z Costell M Fässler R. Integrin Activation by Talin, Kindlin and Mechanical Forces. Nat Cell Biol (2019) 21(1):25–31. doi: 10.1038/s41556-018-0234-9 30602766

[49] Theodosiou M Widmaier M Böttcher RT Rognoni E Veelders M Bharadwaj M. Kindlin-2 Cooperates With Talin to Activate Integrins and Induces Cell Spreading by Directly Binding Paxillin. Elife (2016) 5:e10130. doi: 10.7554/eLife.10130 26821125 PMC4749545

[50] Klapproth S Bromberger T Türk C Krüger M Moser M. A Kindlin-3-Leupaxin-Paxillin Signaling Pathway Regulates Podosome Stability. J Cell Biol (2019) 218(10):3436–54. doi: 10.1083/jcb.201903109 PMC6781449 31537712

[51] Zhu L Liu H Lu F Yang J Byzova TV Qin J. Structural Basis of Paxillin Recruitment by Kindlin-2 in Regulating Cell Adhesion. Structure (2019) 27(11):1686–1697.e5. doi: 10.1016/j.str.2019.09.006 31590942 PMC6894617

[52] Bialkowska K Qin J Plow EF. Phosphorylation of Kindlins and the Control of Integrin Function. Cells (2021) 10(4):825. doi: 10.3390/cells10040825 33916922 PMC8067640

[53] Gao J Huang M Lai J Mao K Sun P Cao Z. Kindlin Supports Platelet Integrin αiibβ3 Activation by Interacting With Paxillin. J Cell Sci (2017) 130(21):3764–75. doi: 10.1242/jcs.205641 PMC6040092 28954813

[54] Kasirer-Friede A Kang J Kahner B Ye F Ginsberg MH Shattil SJ. ADAP Interactions With Talin and Kindlin Promote Platelet Integrin αiibβ3 Activation and Stable Fibrinogen Binding. Blood (2014) 123(20):3156–65. doi: 10.1182/blood-2013-08-520627 PMC4023421 24523237

[55] Das M Ithychanda SS Qin J Plow EF. Migfilin and Filamin as Regulators of Integrin Activation in Endothelial Cells and Neutrophils. PloS One (2011) 6(10):e26355. doi: 10.1371/journal.pone.0026355 22043318 PMC3197140

[56] Bouvard D Aszodi A Kostka G Block MR Albigès-Rizo C Fässler R. Defective Osteoblast Function in ICAP-1-Deficient Mice. Development (2007) 134(14):2615–25. doi: 10.1242/dev.000877 PMC2793408 17567669

[57] Ithychanda SS Qin J. Evidence for Multisite Ligand Binding and Stretching of Filamin by Integrin and Migfilin. Biochemistry (2011) 50(20):4229–31. doi: 10.1021/bi2003229 PMC3097901 21524097

[58] Liu J Das M Yang J Ithychanda SS Yakubenko VP Plow EF. Structural Mechanism of Integrin Inactivation by Filamin. Nat Struct Mol Biol (2015) 22(5):383–9. doi: 10.1038/nsmb.2999 PMC4424056 25849143

[59] Rantala JK Pouwels J Pellinen T Veltel S Laasola P Mattila E. SHARPIN Is an Endogenous Inhibitor of β1-Integrin Activation. Nat Cell Biol (2011) 13(11):1315–24. doi: 10.1038/ncb2340 PMC3257806 21947080

[60] Gao J Bao Y Ge S Sun P Sun J Liu J. Sharpin Suppresses β1-Integrin Activation by Complexing With the β1 Tail and Kindlin-1. Cell Commun Signal (2019) 17(1):101. doi: 10.1186/s12964-019-0407-6 31429758 PMC6700787

[61] Bianconi D Unseld M Prager GW. Integrins in the Spotlight of Cancer. Int J Mol Sci (2016) 17(12):2037. doi: 10.3390/ijms17122037 PMC5187837 27929432

[62] Plaks V Kong N Werb Z. The Cancer Stem Cell Niche: How Essential Is the Niche in Regulating Stemness of Tumor Cells? Cell Stem Cell (2015) 16(3):225–38. doi: 10.1016/j.stem.2015.02.015 PMC4355577 25748930

[63] Lechler T Fuchs E. Asymmetric Cell Divisions Promote Stratification and Differentiation of Mammalian Skin. Nature (2005) 437(7056):275–80. doi: 10.1038/nature03922 PMC1399371 16094321

[64] Lathia JD Gallagher J Heddleston JM Wang J Eyler CE Macswords J. Integrin Alpha 6 Regulates Glioblastoma Stem Cells. Cell Stem Cell (2010) 6(5):421–32. doi: 10.1016/j.stem.2010.02.018 PMC2884275 20452317

[65] Zhu Z Mesci P Bernatchez JA Gimple RC Wang X Schafer ST. Zika Virus Targets Glioblastoma Stem Cells Through a SOX2-Integrin αvβ5 Axis. Cell Stem Cell (2020) 26(2):187–204.e10. doi: 10.1016/j.stem.2019.11.016 31956038 PMC9628766

[66] Xie J Yang P Lin HP Li Y Clementino M Fenske W. Integrin α4 Up-Regulation Activates the Hedgehog Pathway to Promote Arsenic and Benzo[α]Pyrene Co-Exposure-Induced Cancer Stem Cell-Like Property and Tumorigenesis. Cancer Lett (2020) 493:143–55. doi: 10.1016/j.canlet.2020.08.015 PMC9083130 32860851

[67] Murphy DA Courtneidge SA. The 'Ins' and 'Outs' of Podosomes and Invadopodia: Characteristics, Formation and Function. Nat Rev Mol Cell Biol (2011) 12(7):413–26. doi: 10.1038/nrm3141 PMC3423958 21697900

[68] Borghi N Lowndes M Maruthamuthu V Gardel ML Nelson WJ. Regulation of Cell Motile Behavior by Crosstalk Between Cadherin- and Integrin-Mediated Adhesions. Proc Natl Acad Sci USA (2010) 107(30):13324–9. doi: 10.1073/pnas.1002662107 PMC2922157 20566866

[69] Gimond C van der Flier A van Delft S Brakebusch C Kuikman I Collard JG. Induction of Cell Scattering by Expression of Beta1 Integrins in Beta1-Deficient Epithelial Cells Requires Activation of Members of the Rho Family of GTPases and Downregulation of Cadherin and Catenin Function. J Cell Biol (1999) 147(6):1325–40. doi: 10.1083/jcb.147.6.1325 PMC2168093 10601344

[70] Martinez-Rico C Pincet F Thiery JP Dufour S. Integrins Stimulate E-Cadherin-Mediated Intercellular Adhesion by Regulating Src-Kinase Activation and Actomyosin Contractility. J Cell Sci (2010) 123(Pt 5):712–22. doi: 10.1242/jcs.047878 20144995

[71] Ju JA Godet I Ye IC Byun J Jayatilaka H Lee SJ. Hypoxia Selectively Enhances Integrin α5β1 Receptor Expression in Breast Cancer to Promote Metastasis. Mol Cancer Res (2017) 15(6):723–34. doi: 10.1158/1541-7786.MCR-16-0338 PMC5510543 28213554

[72] Shi Y Sun L Zhang R Hu Y Wu Y Dong X. Thrombospondin 4/Integrin α2/HSF1 Axis Promotes Proliferation and Cancer Stem-Like Traits of Gallbladder Cancer by Enhancing Reciprocal Crosstalk Between Cancer-Associated Fibroblasts and Tumor Cells. J Exp Clin Cancer Res (2021) 40(1):14. doi: 10.1186/s13046-020-01812-7 33407730 PMC7789630

[73] Hamidi H Ivaska J. Every Step of the Way: Integrins in Cancer Progression and Metastasis. Nat Rev Cancer (2018) 18(9):533–48. doi: 10.1038/s41568-018-0038-z PMC6629548 30002479

[74] Yue J Zhang K Chen J. Role of Integrins in Regulating Proteases to Mediate Extracellular Matrix Remodeling. Cancer Microenviron (2012) 5(3):275–83. doi: 10.1007/s12307-012-0101-3 PMC3460049 22437309

[75] Munshi HG Stack MS. Reciprocal Interactions Between Adhesion Receptor Signaling and MMP Regulation. Cancer Metastasis Rev (2006) 25(1):45–56. doi: 10.1007/s10555-006-7888-7 16680571

[76] Attieh Y Vignjevic DM. The Hallmarks of CAFs in Cancer Invasion. Eur J Cell Biol (2016) 95(11):493–502. doi: 10.1016/j.ejcb.2016.07.004 27575401

[77] Attieh Y Clark AG Grass C Richon S Pocard M Mariani P. Cancer-Associated Fibroblasts Lead Tumor Invasion Through Integrin-β3-Dependent Fibronectin Assembly. J Cell Biol (2017) 216(11):3509–20. doi: 10.1083/jcb.201702033 PMC5674886 28931556

[78] Erdogan B Ao M White LM Means AL Brewer BM Yang L. Cancer-Associated Fibroblasts Promote Directional Cancer Cell Migration by Aligning Fibronectin. J Cell Biol (2017) 216(11):3799–816. doi: 10.1083/jcb.201704053 PMC5674895 29021221

[79] Labernadie A Kato T Brugués A Serra-Picamal X Derzsi S Arwert E. A Mechanically Active Heterotypic E-Cadherin/N-Cadherin Adhesion Enables Fibroblasts to Drive Cancer Cell Invasion. Nat Cell Biol (2017) 19(3):224–37. doi: 10.1038/ncb3478 PMC5831988 28218910

[80] Knowles LM Gurski LA Engel C Gnarra JR Maranchie JK Pilch J. Integrin αvβ3 and Fibronectin Upregulate Slug in Cancer Cells to Promote Clot Invasion and Metastasis. Cancer Res (2013) 73(20):6175–84. doi: 10.1158/0008-5472.CAN-13-0602 PMC3837455 23966293

[81] Malik G Knowles LM Dhir R Xu S Yang S Ruoslahti E. Plasma Fibronectin Promotes Lung Metastasis by Contributions to Fibrin Clots and Tumor Cell Invasion. Cancer Res (2010) 70(11):4327–34. doi: 10.1158/0008-5472.CAN-09-3312 PMC2880218 20501851

[82] Lambert AW Pattabiraman DR Weinberg RA. Emerging Biological Principles of Metastasis. Cell (2017) 168(4):670–91. doi: 10.1016/j.cell.2016.11.037 PMC5308465 28187288

[83] Paul NR Jacquemet G Caswell PT. Endocytic Trafficking of Integrins in Cell Migration. Curr Biol (2015) 25(22):R1092–105. doi: 10.1016/j.cub.2015.09.049 26583903

[84] Wojnacki J Galli T. Membrane Traffic During Axon Development. Dev Neurobiol (2016) 76(11):1185–200. doi: 10.1002/dneu.22390 26945675

[85] Handorf AM Zhou Y Halanski MA Li WJ. Tissue Stiffness Dictates Development, Homeostasis, and Disease Progression. Organogenesis (2015) 11(1):1–15. doi: 10.1080/15476278.2015.1019687 25915734 PMC4594591

[86] Matsui H Harada I Sawada Y. Src, p130Cas, and Mechanotransduction in Cancer Cells. Genes Cancer (2012) 3(5-6):394–401. doi: 10.1177/1947601912461443 23226577 PMC3513792

[87] Sun Z Guo SS Fässler R. Integrin-Mediated Mechanotransduction. J Cell Biol (2016) 215(4):445–56. doi: 10.1083/jcb.201609037 PMC5119943 27872252

[88] Robinson SD Hodivala-Dilke KM. The Role of β3-Integrins in Tumor Angiogenesis: Context Is Everything. Curr Opin Cell Biol (2011) 23(5):630–7. doi: 10.1016/j.ceb.2011.03.014 21565482

[89] Guo W Pylayeva Y Pepe A Yoshioka T Muller WJ Inghirami G. Beta 4 Integrin Amplifies ErbB2 Signaling to Promote Mammary Tumorigenesis. Cell (2006) 126(3):489–502. doi: 10.1016/j.cell.2006.05.047 16901783

[90] da Silva RG Tavora B Robinson SD Reynolds LE Szekeres C Lamar J. Endothelial Alpha3beta1-Integrin Represses Pathological Angiogenesis and Sustains Endothelial-VEGF. Am J Pathol (2010) 177(3):1534–48. doi: 10.2353/ajpath.2010.100043 PMC2928983 20639457

[91] Huang P Rani MR Ahluwalia MS Bae E Prayson RA Weil RJ. Endothelial Expression of TNF Receptor-1 Generates a Proapoptotic Signal Inhibited by Integrin α6β1 in Glioblastoma. Cancer Res (2012) 72(6):1428–37. doi: 10.1158/0008-5472.CAN-11-2621 PMC3428239 22396498

[92] Wen S Hou Y Fu L Xi L Yang D Zhao M. Cancer-Associated Fibroblast (CAF)-Derived IL32 Promotes Breast Cancer Cell Invasion and Metastasis *via* Integrin B3-P38 MAPK Signalling. Cancer Lett (2019) 442:320e32. doi: 10.1016/j.canlet.2018.10.015 30391782

[93] Uchihara T Miyake K Yonemura A Komohara Y Itoyama R Koiwa M. Extracellular Vesicles From Cancer-Associated Fibroblasts Containing Annexin A6 Induces FAK-YAP Activation by Stabilizing B1 Integrin, Enhancing Drug Resistance. Cancer Res (2020) 80:3222e35. doi: 10.1158/0008-5472.CAN-19-3803 32605995

[94] Peng C Zou X Xia W Gao H Li Z Liu N. Integrin Avb6 Plays a Bi-Directional Regulation Role Between Colon Cancer Cells and Cancer-Associated Fibroblasts. Biosci Rep (2018) 38:BSR20180243. doi: 10.1042/BSR20180243 30355650 PMC6435516

[95] Slack RJ Macdonald SJF Roper JA Jenkins RG Hatley RJD. Emerging Therapeutic Opportunities for Integrin Inhibitors. Nat Rev Drug Discov (2022) 21(1):60–78. doi: 10.1038/s41573-021-00284-4 34535788 PMC8446727

[96] Raab-Westphal S Marshall JF Goodman SL. Integrins as Therapeutic Targets: Successes and Cancers. Cancers (Basel) (2017) 9(9):110. doi: 10.3390/cancers9090110 PMC5615325 28832494

[97] Baiula M Greco R Ferrazzano L Caligiana A Hoxha K Bandini D. Integrin-Mediated Adhesive Properties of Neutrophils Are Reduced by Hyperbaric Oxygen Therapy in Patients With Chronic Non-Healing Wound. PloS One (2020) 15(8):e0237746. doi: 10.1371/journal.pone.0237746 32810144 PMC7433869

[98] Xie B Ren Y Geng J He X Ban C Wang S. Idiopathic Pulmonary Fibrosis Registry China Study (PORTRAY): Protocol for a Prospective, Multicentre Registry Study. BMJ Open (2020) 10(11):e036809. doi: 10.1136/bmjopen-2020-036809 PMC7661367 33177132

[99] Abeysekera KWM Fernandes GS Hammerton G Portal AJ Gordon FH Heron J. Prevalence of Steatosis and Fibrosis in Young Adults in the UK: A Population-Based Study. Lancet Gastroenterol Hepatol (2020) 5(3):295–305. doi: 10.1016/S2468-1253(19)30419-4 31954687 PMC7026693

[100] Zheng S Chen Z Huang C Chen Y Miao W. [99mtc]3PRGD2 for Integrin Receptor Imaging of Esophageal Cancer: A Comparative Study With [18F]FDG PET/Ct. Ann Nucl Med (2019) 33(2):135–43. doi: 10.1007/s12149-018-1315-3 30392070

[101] Sun CC Qu XJ Gao ZH. Arginine-Glycine-Aspartate-Binding Integrins as Therapeutic and Diagnostic Targets. Am J Ther (2016) 23(1):e198–207. doi: 10.1097/MJT.0000000000000053 24621642

[102] Shi J Wang F Liu S. Radiolabeled Cyclic RGD Peptides as Radiotracers for Tumor Imaging. Biophys Rep (2016) 2(1):1–20. doi: 10.1007/s41048-016-0021-8 27819026 PMC5071373

[103] Wu J Wang S Zhang X Teng Z Wang J Yung BC. 18f-Alfatide II PET/CT for Identification of Breast Cancer: A Preliminary Clinical Study. J Nucl Med (2018) 59(12):1809–16. doi: 10.2967/jnumed.118.208637 PMC6910641 29700127

[104] Quigley NG Steiger K Hoberück S Czech N Zierke MA Kossatz S. PET/CT Imaging of Head-and-Neck and Pancreatic Cancer in Humans by Targeting the "Cancer Integrin" αvβ6 With Ga-68-Trivehexin. Eur J Nucl Med Mol Imaging (2021) 24:1–12. doi: 10.1007/s00259-021-05559-x PMC8460406 34559266

[105] Cheng TM Chang WJ Chu HY De Luca R Pedersen JZ Incerpi S. Nano-Strategies Targeting the Integrin αvβ3 Network for Cancer Therapy. Cells (2021) 10(7):1684. doi: 10.3390/cells10071684 34359854 PMC8307885

